# Impact of Education as a Social Determinant on the Risk of Type 2 Diabetes Mellitus in Korean Adults

**DOI:** 10.3390/healthcare12141446

**Published:** 2024-07-19

**Authors:** Mi-Joon Lee, Bum-Jeun Seo, Yeon-Sook Kim

**Affiliations:** 1 Department of Medical Information, Kongju National University, 56 Gongjudaehak-ro, Gongju-si 32588, Republic of Korea; mijoon1004@kongju.ac.kr; 2 Department of Nursing, California State University San Bernardino, San Bernardino, CA 92407, USA; yeon.kim@csusb.edu

**Keywords:** type 2 diabetes mellitus, education, social determinant, health inequity, adult

## Abstract

Education is correlated with health literacy, which is a combination of reading and listening skills, data analysis, and decision-making during the necessary health situations. This study aims to evaluate the effect of education on the risk of type 2 diabetes mellitus (T2DM). This is a population-based cross-sectional study using the 2019 nationwide survey data in Korea. There were 3951 study subjects, after excluding participants with missing data for key exposures and outcome variables. Descriptive statistics, χ^2^ (chi-square) test, and logistic regression were performed to analyze the data. The prevalence of T2DM was associated with educational attainment, sex, age, smoking status, physical activity, carbohydrate intake, and obesity. In the logistic regression model, the odds ratio (OR) of having T2DM was much lower among people educated in college or higher (OR = 0.49, 95% confidence interval [95% CI] = 0.34–0.64) than those with only or without primary education after adjusting for biological factors (sex, age) and health behaviors (smoking status, physical activity, carbohydrate intake, and obesity). This study shows that educational attainment is a significant social determinant influencing health outcomes both directly and indirectly. Therefore, it is necessary to develop policies to reduce the health inequity of T2DM caused by differences in educational attainment.

## 1. Introduction

As health care shifts toward a greater focus on population-driven, evidence-based care, social determinants of health (SDoH) have emerged as essential components to achieve health equity [1]. The detrimental effect of health inequity in disadvantaged communities was highlighted more through the recent COVID-19 pandemic in the U.S. and the Centers for Disease Control and Prevention (CDC) has also published statements on SDoH for nonmedical factors that influence health outcomes; moreover, the SDoH was adopted from the World Health Organization (WHO) [2,3,4,5].

In diabetes, major organizations (for example, the World Health Organization) and professional associations (for example, the American Diabetes Association) have continued to identify health behavior as a major determinant of diabetes prevalence [6,7]. This has led to well-directed studies for underlying genetic and bio-physiological explanations that identified valid biomedical interventions to reduce the risk of diabetes [8,9,10,11,12,13]. In contrast, researchers have also continued to find that socioeconomic status may also be an important determinant of diabetes prevalence, even clarifying the widely accepted education effect in chronic diseases [14,15,16,17,18]. The WHO defined health literacy as “the individual’s ability needed to access, understand, appraise, and use information and services to make appropriate decisions about health” and health literacy is known to be associated with health outcomes, including chronic disease enabling the adoption of a healthy lifestyle, such as consuming balanced diets, engaging in regular physical activity, and maintaining appropriate weight, which are fundamental in diabetes prevention [19,20]. The literacy and numeracy skills to prevent chronic disease such as diabetes are developed through formal education [21].

Diabetes is a major global public health problem, which leads to disability, morbidity, and mortality, and it is rapidly increasing in incidence and prevalence [22]. Furthermore, it was estimated that 537 million people would have diabetes in 2021, and this number is projected to reach 643 million by 2030, and 783 million by 2045 [23]. In Korea, the prevalence of diabetes among adults aged 30 years and older in 2012 increased from 11.8% to 16.7% (about 6.1 million people) in 2020 and diabetes was the sixth leading cause of death among both men and women [24,25]. In South Korea, the economic burden of diabetes was USD 18.3 billion in 2019, which is equivalent to approximately 1.14% of the gross domestic product (GDP) in Korea [26].

Solar and Irwin proposed a framework illustrating the different types of social determinants of health and the causal association between these determinants and health. The WHO Commission on Social Determinants of Health (SDH) adopted the framework to support in prioritizing intervention policies to address healthcare issues effectively and efficiently [27,28].

The WHO conceptual SDH framework demonstrated how socioeconomic and political factors, such as education, gender, income, occupation, race, and ethnicity influence health outcomes playing a role in determining a person’s socioeconomic position [29]. In this framework, social determinants were broadly classified into two main categories that collaborate to influence health and well-being: structural determinants and intermediary determinants. The structural determinants have a socioeconomic and political context producing the social hierarchies through a set of structural mechanisms, including education, gender, income, occupation, race, and ethnicity. The intermediary determinants have a direct impact on people’s health as they determine the vulnerability and exposure to factors that affect people’s health, including psychosocial, behavioral, and biological factors [30].

The purpose of this study was to determine whether educational attainment is associated with type 2 diabetes mellitus (T2DM). To achieve this objective, the first aim was to identify the biological factors and health behaviors associated with T2DM. The second aim was to determine the health characteristics related to educational attainment. The third aim was to quantitatively estimate the impact of educational attainment on the risk of type 2 diabetes after adjusting for confounding variables including biological and health-related factors. To evaluate the health inequities in diabetes, a nationwide large sample from the Korean population was used and the association between educational attainment and the prevalence of diabetes was investigated based on the WHO’s SDH framework. This study not only showed the quantitative relationship between education attainment and the risk of type 2 diabetes mellitus through an unadjusted crude model, but it also used adjusted models for socioeconomic factors such as sex and age, as well as health-related factors such as smoking status, alcohol drinking, physical activity, carbohydrates intake, stress, and BMI in Korean adults aged 30 years and older.

## 2. Materials and Methods

### 2.1. Data

This study used data selected from the Korea National Health and Nutrition Examination Survey (KNHANES) which was conducted by the Korea Center for Disease Control and Prevention (KCDC) and randomly samples approximately 10,000 individuals living in 4800 households chosen annually from the Korean population. The KNHANES used a complex, multi-stage probability sampling method to represent the adult population in South Korea. Initially developed as a periodic survey, it transitioned to an annual survey format in 2007. The sampling process employed three stages, beginning with the selection of primary sampling units (PSUs) from census blocks or resident registration addresses. Each PSU includes about 50–60 households, from which 20 households are chosen for detailed screening. All members aged 1 year and over in these households are then selected for participation. Annually, approximately 10,000 individuals across 192 PSUs are surveyed, with the sample size based on historical response rates from past KNHANES data [31]. KNHANES comprises a health survey, medical examination, and nutritional survey. The health survey collects data on socioeconomic characteristics (age, sex, education, income), health status (morbidity, medical care utilization, physical activities, mental health), and health behaviors (smoking, drinking). The medical examination includes data such as fasting plasma glucose (FPG), blood pressure, and body mass index (BMI) [32]. For this nationwide survey, KCDC formed public–private partnerships with relevant academic societies and approximately 30 expert advisory committees composed of over 180 experts participated in quality assurance and control. Interviews for the health survey and nutrition surveys were conducted through a computer-assisted personal interviewing, which increased the data accuracy by addressing process standardization and decreasing the response burden of participants. Since 1998, the average response rate of each survey was 75.8% [33].

**Figure 1:**
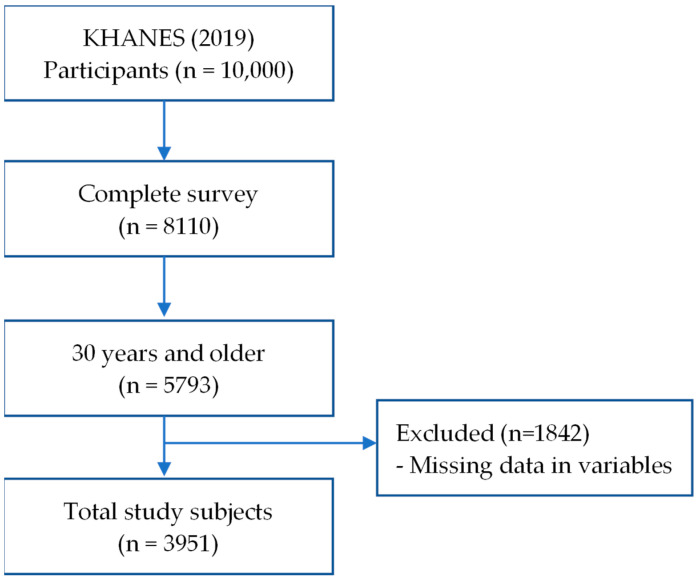
Flow chart for study population selection.

In the KNHANES 2019 survey, a total of 8110 participants successfully completed the health interview, health examination, and nutrition survey. Among these respondents, 5793 were aged 30 years and older. However, 1842 individuals from this group were excluded due to missing data on the variables of interest. Therefore, the final analysis included a total of 3951 individuals (Figure 1).

### 2.2. Model for Health Equity in Type 2 Diabetes Mellitus

**Figure 2:**
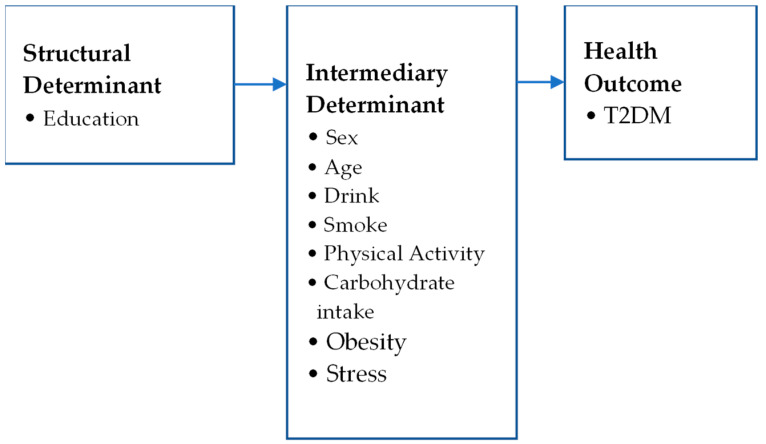
Model for health equity in T2DM.

Figure 2 illustrates the model to evaluate health equity in diabetes. All variables were selected from the KNHANES 2019 dataset corresponding to structural and intermediary factors based on the WHO’s SDH framework. The structural determinant includes education as a social factor affecting the health outcome, diabetes. The intermediary determinants include biological factors, such as sex and age, associated with the prevalence of diabetes, and health behavior factors, such as drinking, smoking, physical activity, carbohydrate intake, obesity, and stress, which are known to influence diabetes [34,35,36,37].

The outcome measure to evaluate health equity is the diagnosis of T2DM, which is defined as FPG ≥ 126 mg/dL, current use of anti-diabetic medication(s), a previous history of diabetes, or HbA1c ≥ 6.5% [38]. In the KNHANES, a blood sample of 8.5 mL (based on a serum separation tube) was collected by a team of experts, and the collected sample was transported while maintaining the refrigerated temperature (2–8 degrees Celsius) through the transport system of a specialized blood testing institution. The specimens that arrived at the testing institution were moved to the testing room, and fasting blood glucose and glycated hemoglobin tests were performed [39].

### 2.3. Structural Determinant

This study examined the education level of participants. Their education level was grouped into no or primary education, middle school, high school, and college or higher education according to their educational attainment [40].

### 2.4. Intermediary Determinants

This study investigated health-related intermediary determinants of participants, such as biological factors (sex, age) and health behavior factors (drinking, smoking, physical activity, obesity, stress, and carbohydrate intake). As a biological factor, sex was di-vided into male and female, and age was grouped into 30–39, 40–49, 50–64, and ≥65 years [41].

As a health behavioral factor, drinking was classified by drinking frequency into occasionally (below 2 times a week) and frequently (more than 2 times a week). Smoking was classified by smoking status into non-smoker, current smoker, and ex-smoker. Physical activity was examined by the walking or running time in a week, which was calculated with the number of days the participant walked or ran for at least ten minutes continuously at any given time during the past week as well as walking or running time in each day. Based on the data, participants were classified into active (more than 150 min a week) and inactive (below 150 min a week) [42]. Carbohydrate intake was measured by the average daily percentage of energy from carbohydrates, and it was divided into two groups for the prevention of diabetes: appropriate amount (less than 60%) and inappropriate amount (more than 60%) [43,44]. For obesity, participants were classified into obese (body mass index in kg/m^2^ [BMI] ≥ 25) and non-obese (BMI < 25), according to the criteria set by the Korean Society for the Study of Obesity [45]. Participants self-reported their stress levels using a questionnaire, categorizing them into four groups: ‘rarely stressed’, ‘slightly stressed’, ‘quite stressed’, and ‘very stressed’. In this study, ‘rarely stressed’ and ‘slightly stressed’ were classified as ‘moderately or less stressed’, while ‘quite stressed’ and ‘very stressed’ were categorized as ‘severely or more stressed’ [46].

### 2.5. Statistical Analysis

To describe socioeconomic and health-related characteristics of the study subjects, descriptive statistics, including mean, frequency, and percentage, were calculated. The χ^2^ (chi-square) test was employed to examine whether there is a difference in the prevalence of T2DM according to the structural determinant (education) and intermediary determinants. Logistic regression analysis was performed to identify factors affecting the risk of T2DM. The aim of epidemiological study is to search for the cause of diseases, and confounding variables or confounders are often defined as the variables affecting (positively or negatively) both the dependent variable and the independent variable. Thus, it is necessary to eliminate the effect from confounders being studied so that the results do not reflect the actual relationship between the dependent variable and the independent variable under study. The diagnosis of T2DM was the dependent variable. Education was the independent variable and confounding variables included sex, age, drinking, smoking, physical activity, obesity, and stress. To examine how the risk (odds ratio) of T2DM, the dependent variable, changes based on the independent variable of individual educational attainment, the ‘Enter’ method was employed for logistic regression analysis. In the first stage, only the educational attainment variable was entered. In the second stage, biological covariates identified in previous studies as influencing T2DM were included. In the third stage, health-related covariates were additionally entered. This stepwise approach allowed us to estimate the odds ratio by educational attainment after removing the confounding effects of other covariates. Goodness of fit for the logistic regression model was tested using Hosmer and Lemeshow statistics at the significance level of 0.05 [47]. All data were statistically analyzed using IBM SPSS Statistics version 27.0 (IBM Corp., Armonk, NY, USA). The study protocol received approval from the Institutional Review Board of K National University, and the need for informed consent was waived (reference No. KNU_IRB_2023-010).

## 3. Results

### 3.1. Prevalence of T2DM According to Biological Factors and Health Behaviors

Table 1 shows the characteristics of subjects including their educational attainment and T2DM. Among 3951 participants, 18.1% had no or primary education, 54.6% were female, and 32.9% were aged 50 to 64 years old. A total of 76.1% drank less than two times a week and 56.5% have never smoked. A total of 58.2% performed physical activity for less than 150 min per week. A total of 59.9% have consumed carbohydrates as more than 60% of their daily percentage of energy. A total of 34.7% were obese with a BMI over 25 kg/m^2^. A total of 74.9% were moderately or less stressed and 9.9% had T2DM.

It was also shown that the prevalence of T2DM was higher in men than in women (12.3% vs. 7.9%). The prevalence was highest in people with no or primary education (20.9%), aged 65 years and older (21.3%), and who were ex-smokers (13.7%). The prevalence was higher in physically inactive people than active (10.7% vs. 8.7%), in those with higher carbohydrate intake than less (12.2% vs. 6.4%), and in those with obesity than those non-obese (14.5% vs. 7.4%). Except for drinking status and stress, all socioeconomic and health-related covariates differed in statistical significance in the presence or absence of T2DM (Table 1).

**Figure 3:**
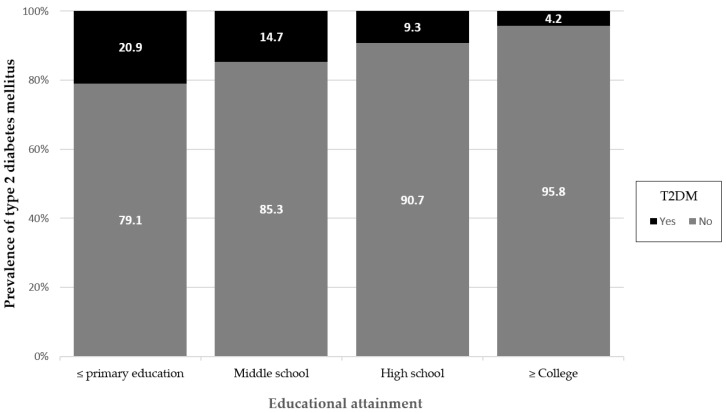
Prevalence of T2DM by educational attainment.

Figure 3 illustrates the relationship between T2DM and the level of education the study subjects received. The prevalence of T2DM was highest in those with no or primary education (20.9%) followed by those with middle school (14.7%), high school (9.3%), and college or higher education (4.2%).

### 3.2. Comparison of Educational Attainment by Health-Related Characteristics

Table 2 shows health-related characteristics in different educational attainments. More women (21.0%) had no or primary education than in men (14.7%). Those with no or primary education included people aged 65 years and older (52.3%) as well as people who never smoked (20.5%). They showed more people drinking less than those drinking more (18.8% vs. 16.1%), more physically inactive than active (22.1% vs. 12.6%), more people with high carbohydrate intake than low intake (24.3% vs. 9.0%), higher obesity than non-obesity (20.8% vs. 16.7%), and higher in moderate or less stress than severe or more stress (18.7% vs. 16.4%). All health-related covariates differed in statistical significance by the level of educational attainment.

### 3.3. Logistic Regression Analysis for the Effect of Education on the Risk of T2DM

To estimate the association between T2DM and education, logistic regression analysis was performed excluding covariates unrelated to the prevalence of T2DM, such as drinking and level of stress. In logistic regression analysis, the crude model includes only the primary independent variable without adjusting for any other factors to examine how the independent variable affects the outcome measure. The adjusted model, on the other hand, adjusts for additional covariates or confounders to account for their potential influence on the relationship between the independent and dependent variables. To separately estimate the effects of biological covariates and health behavior covariates, we sequentially included these variables in the adjusted model. Table 3 shows that education is associated with the risk of T2DM. In the crude model, the odds ratios of T2DM were relatively lower in those who had college or higher education (OR = 0.17, *p* < 0.001), high school education (OR = 0.39, *p* < 0.001), and middle school education (OR = 0.65, *p* = 0.011) compared to those who had no or primary education. The odds ratios of T2DM were statistically significant. After adjusting biological covariates such as sex and age, the odds ratios of T2DM were still lower in those who had at least a college education (OR = 0.45, *p* < 0.001) and high school education (OR = 0.68, *p* = 0.012) compared to those who attained no or primary education. Those who had middle school education also showed a lower risk of T2DM (OR = 0.77, *p* = 0.132) but it was not statistically significant. In the final model, education still showed a significant association with the risk of T2DM in those who had at least a college education (OR = 0.49, *p* < 0.001) and high school education (OR = 0.73, *p* = 0.043) compared to those who attained no or primary education when it was further adjusted for health behavior covariates including smoking, physical activity, carbohydrate intake, and obesity. Drinking and stress were excluded from the final model as they did not show a significant association with T2DM in Table 1. All three logistic regression models demonstrated a goodness of fit based on the Hosmer–Lemeshow test.

## 4. Discussion

This is a cross-sectional study using a large scale of nationwide population data in Korean adults. This study showed that 21.3% of Korean older adults aged 65 years and older had T2DM, which was similar to the prevalence of 20.5% in the U.S. and 19.3% in the world [48,49]. This study showed that 28.2% of Korean adults did not complete high school education, which was a little higher than the rate in the U.S. (20.4%) [50].

This study showed that the risk of T2DM increased as the educational attainment of people decreased. This gradient of education is consistent with the findings of other precious studies [51,52]. The previous studies which only used self-reported cases as the outcome of diabetes are less reliable than the medically examined cases obtained in this study as the use of self-report can underestimate the prevalence of diabetes because of individuals who have been diagnosed but are not responding [53,54]. Moreover, the effect of these issues on the under-representation of diabetes patients was most commonly observed in low-educated people [55]. Our findings stating that educational attainment is associated with the prevalence of T2DM provide rationales for establishing the health policy for those with low education who have diabetes but are not aware of it due to a lack of knowledge of the disease. At the individual level, educational achievement affects an individual’s health knowledge, attitudes, and behaviors. Higher education levels are often associated with better health literacy, which can lead to healthier lifestyle choices such as proper diet and regular physical activity, which are important for preventing T2DM. Furthermore, on a social level, education can affect socioeconomic status, which in turn can affect healthcare access, employment status, and residential environment. Higher socioeconomic status often provides more access to healthcare for early detection and management of T2DM and reducing exposure to stressors associated with lower socioeconomic conditions [56]. Accordingly, this study may potentially contribute to decreasing the health inequity of diabetes.

Consistent with previous studies, this study showed that the prevalence of T2DM was associated with biological characteristics, including sex and age [57,58]. These findings suggest that strategies to maintain adequate diabetes prevention programs should be developed based on the biological characteristics of individuals.

This study showed that ex-smokers or current smokers had a higher prevalence of T2DM than people who never smoked, and it is similar to the previous study indicating that smoking increases inflammation in the body and causes oxidative stress incurring damage to cells. Both inflammation and oxidative stress may be related to an increased risk of diabetes [59]. In this study, the prevalence of T2DM was higher in physically inactive people who have walked or run for under 150 min a week than in physically active people, which is consistent with the results of previous studies indicating an inverse association between physical activity and the onset of diabetes [60,61]. It was shown that carbohydrate intake and obesity were significantly associated with T2DM in this study, and these findings are in line with the previous study that prevention of diabetes can be accomplished through weight loss with intensive lifestyle interventions that include caloric reduction [62]. According to these findings, we suggest that intervention programs should incorporate smoking cessation, weight loss, and physical activity to mitigate the risk of T2DM.

The study revealed a significant association between low educational attainment and an elevated risk of T2DM and the association remained statistically significant after adjusting for biological factors. As the effect estimates in the models are based on retrospective data, there might have been biases and confounding variables influencing the estimates of effect. Thus, the strength and direction of the association between T2DM and education were estimated by adjusting covariates to reflect the confounding effect. The association was still significant when it was further adjusted for health behavior factors. Individuals with higher educational attainment are able to access informational sources well and implement adequate health behaviors such as nutrition intake and physical activity [63]. Therefore, it is assumed that educational attainment affects the prevalence of disease according to the knowledge strongly influencing people’s ability to prevent or better manage T2DM after being diagnosed. Accordingly, the findings of this study emphasize the significance of education when establishing health strategies and policies aimed at managing the T2DM risk in Korean adults.

This study has potential limitations. First, it should be noted that the association between the diagnosis of T2DM and educational levels was not estimated in different age groups. The risk of T2DM may be lower or higher at a younger age, but the risk may also be diluted or intensified in old people [64]. Second, this study used self-reported survey data, and education level was also self-reported, which may not accurately reflect actual educational attainment of each subject and, for other variables as well, there could be a higher likelihood of measurement errors compared to experimental studies that use pragmatic methods for variable measurement. Educational attainment was categorized based on the duration of education, thus overlooking the capacity of individual educational institutions and regional characteristics. Furthermore, since educational attainment is not a direct indicator of an individual’s health literacy, future research should directly measure health literacy to analyze its impact on the risk of T2DM. Third, the risk of T2DM can be influenced not only by social factors such as education, gender, income, occupation, race, and ethnicity, but also by factors like accessibility to fresh foods and exposure to marketing of fast food [65]. Therefore, it is necessary to explore the risk of type 2 diabetes onset associated with food accessibility in follow-up studies in this area. In this study, the measured daily carbohydrate intake does not distinguish between consumption from composite foods and consumption from single foods, indicating a need for a next study to include differentiation based on the food intake method. Forth, the ‘stress’ measured in this study was based on self-reported questionnaires, which presents a limitation as it does not account for the variability in stress due to individual environmental factors such as family status, location, financial supporters, and illness. Fifth, while this study considered BMI as a factor influencing T2DM, recent studies have actively explored more refined measures such as body fat mass and muscle mass, providing a more detailed understanding [66]. Sixth, since this study used one year of KHNANES data, it is necessary to use multiple years of data to improve statistical power in a further study. Accordingly, this study used a cross-sectional design in which both the exposure and outcome are assessed simultaneously. Seventh, comorbidities such as hypertension or family history of diabetes are also significant factors influencing T2DM [67,68]. Therefore, it is recommended that a next study includes comorbidities or family history as covariates in the analysis. As a consequence, the association between education and the T2DM risk cannot be definitively interpreted as the causality.

## 5. Conclusions

In conclusion, this study shows that educational attainment is a significant social determinant influencing health outcomes both directly and indirectly. The knowledge and skills attained through education may influence a person’s cognitive function, make them more perceptive to health education contents, or enable them to access appropriate health services and communicate better. Therefore, it is recommended to conceptualize the role of education in health outcomes, particularly in terms of knowledge, cognitive skills, and analytical abilities. Thus, it is necessary to develop policies to reduce the health inequity in diabetes caused by differences in educational environment. This study also indicated that a history of smoking and excessive carbohydrate intake increase the risk of type 2 diabetes mellitus. Therefore, a further study on effective health management strategies and tools is necessary to help individuals better manage their health behaviors.

## Figures and Tables

**Table 1:** Prevalence of T2DM by socioeconomic and health-related characteristics (N = 3951).

Variable	Total	T2DM		χ^2^	*p*-Value
		Yes	No		
	*n* (%)				
Education level				166.733 ***	<0.001
≤Primary education	717 (18.1)	150 (20.9)	567 (79.1)		
Middle school	395 (10.0)	58 (14.7)	337 (85.3)		
High school	1231 (31.2)	114 (9.3)	1117 (90.7)		
≥College	1608 (40.7)	68 (4.2)	1540 (95.8)		
Sex				21.137 ***	<0.001
Male	1794 (45.4)	220 (12.3)	1574 (87.7)		
Female	2157 (54.6)	170 (7.9)	1987 (92.1)		
Age ^1^	00 ± 0.00			251.441 ***	<0.001
30–39	718 (18.2)	8 (1.1)	710 (98.9)		
40–49	900 (22.8)	34 (3.9)	866 (96.1)		
50–64	1301 (32.9)	128 (9.8)	1173 (90.2)		
≥65	1032 (26.1)	220 (21.3)	812 (78.7)		
Drinking				0.030 *	0.821
<2 times/week	3005 (76.1)	298 (9.9)	2707 (90.1)		
≥2 times/week	946 (23.9)	92 (9.7)	854 (90.3)		
Smoking				24.844 ***	<0.001
Never	2232 (56.5)	179 (8.0)	2053 (92.0)		
Ex-smoker	1046 (26.5)	142 (13.6)	904 (86.4)		
Current smoker	673 (17.0)	69 (10.3)	604 (89.7)		
Physical activity ^2^				4.755 *	0.024
≥150 min/week	1653 (41.8)	143 (8.7)	1510 (91.3)		
<150 min/week	2298 (58.2)	247 (10.7)	2051 (89.3)		
Carbohydrate intake ^3^				36.417 ***	<0.001
<60%	1585 (40.1)	101 (6.4)	1484 (93.6)		
≥60%	2366 (59.9)	289 (12.2)	2077 (87.8)		
Obesity ^4^				50.897 ***	<0.001
No (25 > BMI)	2580 (65.3)	191 (7.4)	2389 (92.6)		
Yes (25 ≤ BMI)	1371 (34.7)	199 (14.5)	1172 (85.5)		
Stress				1.230	0.245
Moderate or less	2958 (74.9)	301 (10.2)	2657 (89.8)		
Severe or more	993 (25.1)	89 (9.0)	904 (91.0)		

* *p* < 0.05, *** *p* < 0.001. ^1^ Units expressed as years. ^2^ Units expressed as minutes. ^3^ Units expressed as a percentage of energy from carbohydrate intake per day. ^4^ Units expressed as kg/m^2^.

**Table 2:** Comparison of educational attainment by health-related characteristics (N = 3951).

Variable	Educational Attainment, *n*(%)				χ^2^
	≤Primary Education	Middle School	High School	≥College	
Sex					29.946 ***
Male	264 (14.7)	188 (10.5)	556 (31.0)	786 (43.8)	
Female	453 (21.0)	207 (9.6)	675 (31.3)	822 (38.1)	
Age					182.939 ***
30–39	8 (1.1)	11 (1.5)	155 (21.6)	544 (75.8)	
40–49	6 (0.7)	26 (2.9)	306 (34.0)	562 (62.4)	
50–64	163 (12.5)	184 (14.1)	558 (42.9)	396 (30.4)	
≥65	540 (52.3)	174 (16.9)	212 (20.5)	106 (10.3)	
Drinking					8.997 *
<2 times/week	565 (18.8)	285 (9.5)	916 (30.5)	1239 (41.2)	
≥2 times/week	152 (16.1)	110 (11.6)	315 (33.3)	369 (39.0)	
Smoking					46.462 ***
Never	457 (20.5)	201 (9.0)	653 (29.3)	921 (41.3)	
Ex-smoker	177 (16.9)	118 (11.3)	312 (29.8)	439 (42.0)	
Current smoker	83 (12.3)	76 (11.3)	266 (39.5)	248 (36.8)	
Physical activity					70.179 ***
≥150 min/week	209 (12.6)	146 (8.8)	548 (33.2)	750 (45.4)	
<150 min/week	508 (22.1)	249 (10.8)	683 (29.7)	858 (37.3)	
Carbohydrate intake					217.619 ***
<60%	142 (9.0)	124 (7.8)	496 (31.3)	823 (51.9)	
≥60%	575 (24.3)	271 (11.5)	735 (31.1)	785 (33.2)	
Obesity					20.456 ***
No (25 > BMI)	432 (16.7)	244 (9.5)	794 (30.8)	1110 (43.0)	
Yes (25 ≤ BMI)	285 (20.8)	151 (11.0)	437 (31.9)	498 (36.3)	
Stress					14.439 **
Moderate or less	554 (18.7)	317 (10.7)	926 (31.3)	1161 (38.2)	
Severe or more	163 (16.4)	78 (7.9)	305 (30.7)	447 (45.0)	

* *p* < 0.05, ** *p* < 0.01, *** *p* < 0.001.

**Table 3:** Logistic regression analysis for the effect of education on the risk of T2DM (N = 3951).

	Crude Model ^1^			Adjusted Model 1 ^2^			Adjusted Model 2 ^3^		
Variable	OR	95% CI		OR	95% CI		OR	95% CI	
		Min	Max		Max	Max		Min	Max
Education level									
≤Primary education	1.00			1.00			1.00		
Middle school	0.65 *	0.47	0.91	0.77	0.54	1.08	0.78	0.55	1.11
High school	0.39 ***	0.30	0.50	0.68 *	0.50	0.92	0.73 *	0.53	0.99
≥College	0.17 ***	0.12	0.23	0.45 ***	0.31	0.64	0.49 ***	0.34	0.72
Sex									
Male				1.00			1.00		
Female				0.59 ***	0.47	0.74	0.77	0.55	1.07
Age									
30–39				1.00			1.00		
40–49				3.37 **	1.55	7.34	3.24 **	1.48	7.07
50–64				7.70 ***	3.70	16.03	7.43 ***	3.56	15.52
≥65				14.70 ***	6.98	30.97	14.73 ***	6.92	31.33
Smoking									
Never							1.00		
Ex-smoker							1.45 *	1.02	2.04
Current smoker							1.42	0.97	2.09
Physical activity									
≥150 min/week							1.00		
<150 min/week							1.02	0.81	1.28
Carbohydrate intake									
<60%							1.00		
≥60%							2.10 ***	1.68	2.61
Obesity									
No (25 > BMI)							1.00		
Yes (25 ≤ BMI)							1.28	0.99	1.65
Cox and Snell R^2^/Nagelkerke’s R^2^	0.039/0.083			0.072/0.152			0.084/0.177		
χ^2^(df), *p*-value	158.364(3), <0.001			296.787(7), <0.001			347.219(12), <0.001		
Hosmer–Lemeshow test, χ^2^(df), *p*-value	0.000(2), 1.000			6.765(8), 0.562			13.377(8), 0.100		

* *p* < 0.05, ** *p* < 0.01, *** *p* < 0.001. OR: odds ratio for T2DM. CI: confidence interval. ^1^ Crude model: includes only education. ^2^ Adjusted model 1: adjusted for biological covariates including sex and age. ^3^ Adjusted model 2: adjusted for biological and health-related covariates including sex, age, smoking, physical activity, carbohydrate intake, and obesity.

## Data Availability

All original data are publicly available free of charge from the KNHANES website (http://knhanes.cdc.go.kr) for the purposes of academic research.
